# Survival following complete resection of neuroblastoma in novel orthotopic rat xenograft model

**DOI:** 10.1038/s41598-023-47537-3

**Published:** 2023-11-18

**Authors:** ReidAnn E. Sever, Lauren Taylor Rosenblum, Miguel Reyes-Múgica, W. Barry Edwards, Marcus M. Malek, Gary Kohanbash

**Affiliations:** 1 Department of Neurological Surgery, University of Pittsburgh, 530 45th Street, Pittsburgh, PA 15201 USA; 2 Department of Surgery, University of Pittsburgh, 200 Lothrop Street, Pittsburgh, PA 15213 USA; 3 Department of Pathology, UPMC Children’s Hospital of Pittsburgh, One Children’s Hospital Drive, 4401 Penn Ave, Pittsburgh, PA 15224 USA; 4 Department of Biochemistry, University of Missouri, 117 Schweitzer Hall, Columbia, MO 65211 USA; 5 Department of Pediatric General Surgery, University of Pittsburgh, One Children’s Hospital Drive, 4401 Penn Ave, Pittsburgh, PA 15224 USA; 6 Department of Immunology, University of Pittsburgh Medical Center, 200 Lothrop Street, Pittsburgh, PA 15213 USA

**Keywords:** Cancer, Oncology

## Abstract

Neuroblastoma accounts for 15% of pediatric cancer deaths, despite multimodal therapy including surgical resection. Current neuroblastoma rodent models are insufficient for studying the impact of surgery and combination treatments, largely due to the small size of mouse models. Human neuroblastoma SK-N-BE(2) cells were injected into the left adrenal gland of 5–6-week-old RNU homozygous nude rats. Rats were either monitored by MRI until humane endpoint was reached or after 5 weeks underwent operative tumor resection, followed by monitoring for recurrence and survival. Following neuroblastoma cell implantation, the majority of tumors grew to greater than 5000 mm^3^ within 5.5–6.5 weeks, meeting the humane endpoint. Surgical resection was successfully done in 8 out of 9 rats, extending survival following tumor implantation from a median of 42 days to 78 days (p < 0.005). Pathology was consistent with human neuroblastoma, showing small round blue cell tumors with Homer-Wright rosettes, high mitoses and karyorrhectic index, and strong PHOX2B staining. Thus, we have established a novel orthotopic xenograft rat model of neuroblastoma and demonstrated increased survival of rats after surgical tumor resection. This model can be used for the development of surgical techniques, such as the use of intraoperative molecular imaging or assessment of combination therapies that include surgery.

## Introduction:

Neuroblastoma, a malignancy of neural crest cell origin, is the most common solid extracranial tumor in children^1,2^. Despite developments in chemotherapy, immunotherapy, and radiation treatments, patients with high-risk disease only have a 5-year survival rate of 40–50%^3^. Multimodal treatment includes surgical resection, though encasement of neurovascular structures, unclear tumor margins, and remote deposits of satellite lesions pose significant challenges to complete resection. In fact, the rate of inadequate resection nears 30%, which has important implications as completeness of resection has been shown to affect patient outcomes in multi-center, multi-national studies from the Children’s Oncology Group (COG) and International Society of Pediatric Oncology (SIOP)^4,5^. Furthermore, up to 50% of patients experience complications after surgery^6^. However, current animal models of neuroblastoma are inadequate to assess developments in surgical technique. While survival after resection has been demonstrated in a mouse model, the small size of the abdominal cavity limits tumor growth and as a result, also limits the comparison to human disease^7^. The impact of resection using novel intraoperative contrast agents, for example, would not be feasible in the small mouse model^8^. Therefore, there is a present need to create a larger model of neuroblastoma, particularly to study surgical innovations.

Rat models offer multiple advantages over mouse models of human disease. Most obvious, their larger size (RNU rats with average weight of 175 g are about 8 times larger than Nu/j mice with an average weight of 20 g), enables more delicate operative interventions and blood sampling with less impact on the animal. Rat models enable the growth of larger tumors and better study of subtle changes in tumor size and heterogeneity within a tumor. The longer lifespan also enables easier detection of significant differences in lifespan and time to tumor recurrence. Though they share a common evolutionary branchpoint, rats are more genetically similar to humans than mice: rats have 21 pairs of chromosomes while mice have 20, rats have more intrachromosomal than interchromosomal rearrangements while mice have the reverse^9^. Furthermore, pharmacology and toxicology experiments are more easily and more commonly performed in rats^10,11^.

There are limited rat models of neuroblastoma. Nilsson et al. described subcutaneous flank injections of SH-SY5Y human neuroblastoma cells in RNU rats^12^. Other subcutaneous xenograft models include a LAN-1 model and an SK-N-AS human neuroblastoma^13,14^. The location of the xenograft in the subcutaneous tissue rather than the adrenal gland does not sufficiently replicate the anatomy of the disease for surgical studies and the tumor growth and response might be different in each environment. Adrenal-based xenografts are important not only to replicate the disease more closely, but as a model for surgical resection of the tumor in its most common location.

Engler et al. created NB xenografts with LAN-1 human neuroblastoma cell injections into the tail vein, left adrenal gland, or aorta of 4-week-old RNU rats^15^. Tumors grew rapidly after left adrenal gland injections (25,132 ± 1570 mm^3^ in 5 w), with smaller adrenal tumors in the aorta-injected group (5300 ± 1300 mm^3^) and tiny tumors after tail vein injection (42 ± 12 mm^3^). Furthermore, in rats with intra-aortal injections, 50% had liver micrometastases and 60% had femur micrometastases (as seen with anti-GD2 staining). To our knowledge, however, that model was last described over 20 years ago, and no intra-adrenal rat xenograft model has been created or used since. Furthermore, the MYC-N amplified SK-N-BE(2) neuroblastoma cell line has become an established cell line for xenograft tumors in mice and were derived from the bone marrow of a 2-year-old with disseminated disease who had undergone multiple courses of chemotherapy and radiation^16–18^.

Here, we report on the generation and characterization of a novel adrenal xenograft neuroblastoma model in rats that closely resembles human disease, is amenable to surgical excision, and allows for the study of the effect of surgery on tumor burden, animal survival, and the impact of treatments when combined with resection.

## Materials and methods

### Cell lines

SK-N-BE(2) (ATCC: no. CRL-2271) cells were cultured in MEM alpha (Lonza, Cat #12-349-015), supplemented with heat-inactivated FBS (Hyclone, Cat# SH3091003), sodium pyruvate (Lonza, Cat# BW13-115E), Antibiotic–Antimycotic (Gibco, Cat#15-240-062), beta-mercaptoethanol (Gibco, Cat#21-985-023), MEM NEAA (Gibco, Cat#11140-050), and 50 mg/mL Normocin (Invivogen, Cat# ant-nr-2) and maintained in a 37 °C humidified incubator with 5% CO_2_.

### Animal studies

Animal studies were performed in accordance with the protocols approved by the Institutional Animal Care and Use Committee of the University of Pittsburgh, and in accordance with the relevant guidelines and regulations (IACUC protocol number: 22101912 and ARRIVE guidelines). Five-to-six-week-old female athymic nude rats (nu/nu) (NIH-Foxn1^rnu^, strain code 316), purchased from Charles Rivers Laboratories, were maintained in a temperature-controlled animal facility at the UPMC Children’s Hospital of Pittsburgh with a 12-h light/dark cycle. Animals were kept in the facility for at least two days prior to performing any procedures. Animals were monitored every other day and weighed at least weekly. Humane endpoint was defined as a loss of more than 20% of the original body weight; signs of significant distress from tumor burden such as decreased activity, inability to walk normally, substantially distended abdomen, or body condition score less than 2; or tumor size greater than 4 cm in its largest dimension or 5000 mm^3^ in volume as seen on MRI (per direction by the University of Pittsburgh veterinary group and American Veterinary Medical Association (AVMA) Guidelines for the Euthanasia of Animals (2020))^19^. Animals were euthanized in a carbon dioxide chamber followed by confirmatory cervical dislocation within 12 h of reaching humane endpoint (per approved IACUC protocol in accordance with Human Euthanasia practices).

### Tumor grafts

**Figure 1:**
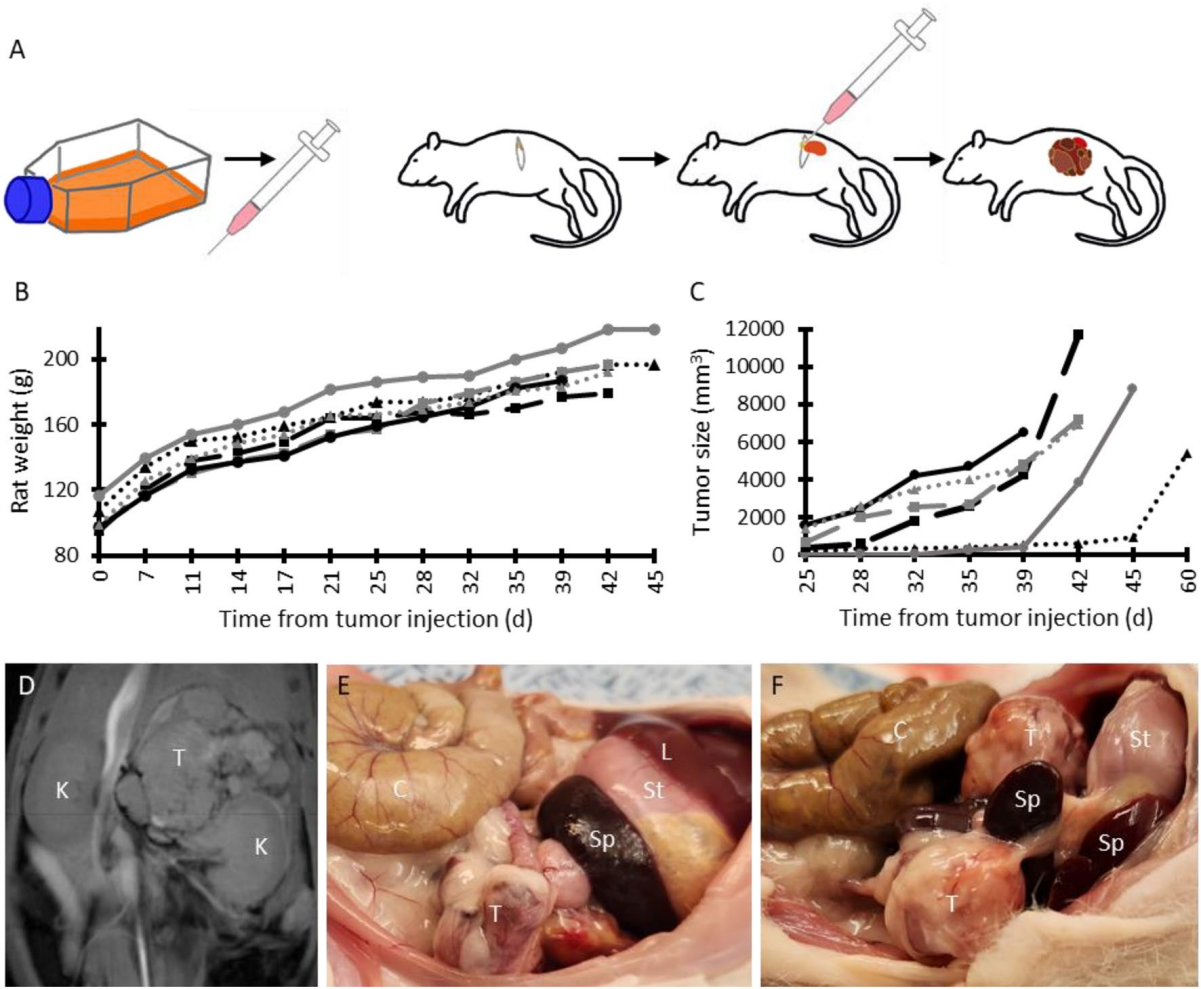
(**A**) Schematic of neuroblastoma implantation: SK-N-BE(2) cells in Matrigel are injected in the left adrenal gland, followed by orthotopic tumor growth. (**B**) Growth of rats following tumor injection (where each line pattern represents a different rat in the survival group). (**C**) Growth of left adrenal tumor starting 25 days after tumor injection. (**D**) Representative coronal MRI of neuroblastoma in rat 5.5 weeks post-injection demonstrating a heterogeneous tumor (T) abutting the left kidney (K). (**E**,**F**) Examples of primary (**E**) and recurrent (**F**) tumors in situ (lateral view with head towards image right; T is tumor, C is colon, Sp is spleen, St is stomach, and L is liver).

Tumors were grafted as previously described in mice, but in 5–6-week-old female RNU homozygous rats (Charles Rivers Laboratories)^20^. SK-N-BE(2) neuroblastoma cells at 70–90% confluence were trypsinized, resuspended in PBS, and counted (10µL mixed with Trypan blue and counted with automated hemocytometer). 1.9 × 10^6^ SK-N-BE(2) neuroblastoma cells in 30 µL of PBS were mixed with 30 µL of Matrigel basement membrane matrix (Corning Cat#354234; Fig. 1A), and pipetted into a 1 cc syringe with a 23-gauge needle for each rat. Under isoflurane anesthesia (using SomnoFlow machine), each rat was placed in right lateral decubitus position with a small bump (i.e. folded gauze) under the upper abdomen, hair was trimmed if necessary, a transverse left flank incision was made through the skin and abdominal wall muscle, and the left kidney and adrenal gland were exteriorized with gentle external pressure and a sterile cotton tip applicator (Fig. 1A). Matrigel-suspended cells were then implanted into the left adrenal gland and surrounding fat pad before the needle was slowly removed (Fig. 1A). The kidney and adrenal gland were returned to the abdominal cavity, the body wall was closed with 3–0 or 4–0 Polysorb suture, and the skin was closed with surgical wound clips. The animals were given meloxicam for analgesia, recovered in a clean cage on a heating pad, and were monitored until awake and active. Wound clips were removed 10–14 days later. Rats that did not have appreciable tumors within 6.5 weeks were removed from further analysis (1 out of 6 rats in the survival group and 3 out of 12 rats in the surgery group).

### Survival and MRI

Following tumor injection, 5–6-week-old rats (n = 6, no surgery cohort) were monitored for growth, symptoms, and tumor development. MRIs were done twice weekly, beginning 3.5 weeks after tumor implantation. Due to the intraperitoneal location of the tumors, size could not be measured externally. Rats were euthanized when the humane endpoint was reached (see “Animal studies” section). One rat did not have an appreciable tumor on MRI 6.5 weeks after implantation and was excluded from further analysis. For the surgery cohort (n = 9), MRIs were performed 5 weeks after tumor injection to confirm adequate growth prior to surgery, then again when rats exhibited symptoms of recurrence.

To obtain each MRI, rats were anesthetized via a nose cone with 1–2% isoflurane and 3L/m O_2_, positioned on an animal bed, and placed in the scanner. Respiration rate was monitored, and body temperature was maintained using a warm air heating system, (SA Instruments, New York, NY, USA). MRI was performed using a 7 T/30-cm AVIII spectrometer (Bruker Biospin, Billerica, MA) equipped with a 12 cm gradient set and using an 86 mm quadrature RF volume coil and Paravision 6.0.1. Anatomical scans on the abdomen were performed using a self-navigating Intragate sequence with retrospective gating on the respiration with the following parameters: repetition time (TR)/echo time (TE) = 200/2.6, field of view (FOV) = 70 × 70 mm, acquisition matrix = 256 × 256, 19 coronal slices with a slice thickness of 1.5 mm. Each scan was performed over a period of 25 min.

### Tumor resection (surgery)

Neuroblastoma tumors were implanted in 5–6-week-old rats (n = 12), as described above. MRI was done 5 weeks after injection and resection was attempted in rats with tumors greater than 0.7 cm in largest dimension (n = 9). Tumor volume was calculated from MRI images using DSI studios DICOM analysis. Three rats did not have appreciable tumors at this time and were excluded from further analysis.

Under 2–3% isoflurane anesthesia and 3L/m O_2_ (administered by a research assistant), the rat was laid supine while respirations were monitored, and body temperature was maintained using a heating pad. A lateral transverse abdominal incision was made (Fig. 3A) and sharp dissection combined with a fine-tip cautery tool (BVI Accu Temp Cat# D901) was used to open the abdominal wall and meticulously separate the tumor from the surrounding healthy tissue. Surgical loupes were typically used by the surgeon for additional magnification. Additional tumor nodules were resected when observed. The peritoneum was closed with 3–0 or 4–0 suture and skin was closed with skin clips, which were removed 10–14 days after surgery. Topical lidocaine spray was sprayed over the incision and 1 mg/kg of meloxicam was given via intraperitoneal injection. Rats did not show signs of pain following surgery nor in the following days. Rats were followed for tumor recurrence and were euthanized when the humane endpoint was reached.

### Histopathological analysis

Tumor samples were fixed in 10% neutral buffered formalin, paraffin-embedded, cut, and stained with hematoxylin and eosin^21^. Immunohistochemical staining for PHOX2B was performed on the Ventana Benchmark Ultra automated staining platform. Slides were pretreated with ultraCC1 (proprietary, Roche Tissue Diagnostics, Indianapolis, IN) and stained using a recombinant antibody to PHOX2B (EPR14423) (Abcam, Cambridge, MA, ab183741). OptiView DAB detection kit (proprietary, indirect, biotin free system, Roche Tissue Diagnostics, Indianapolis, IN) was used for primary antibody detection. All slides were counterstained with hematoxylin and routinely dehydrated, cleared, and cover-slipped in resinous mounting medium.

### Statistical analysis

All statistical analyses were performed with GraphPad Prism (version 10.0.2 for Windows, GraphPad Software, Boston, MA). Tumor sizes were compared using an unpaired t test. Kaplan–Meier analysis with log-rank Mantel-Cox test was used to compare survival of untreated neuroblastoma model rats and neuroblastoma model rats that underwent surgical resection. Simple linear regression was used to assess correlation between tumor volumes on MRI and by caliper measurement, as well as resected and recurrent tumor sizes.

### Ethics approval and consent to participate

Animal studies were performed in accordance with the protocols approved by the Institutional Animal Care and Use Committee of the University of Pittsburgh (IACUC protocol number: 22101912).

## Results

### Generation and characterization of orthotopic xenograft rat model

Here, we demonstrate a novel SK-N-BE(2) human neuroblastoma cell xenograft in the adrenal gland of RNU homozygous rats. Cells suspended in Matrigel were injected directly into the adrenal glands of 5–6-week-old nude rats (n = 5, not including rat which grew a delayed tumor; Fig. 1A). The rats continued to grow following tumor injection (101 ± 8.6 g) until meeting the humane endpoint (195 ± 14.7 g after 42 days), including in the final week (Fig. 1B).

Tumors were visible on MRI beginning 3–5 weeks after tumor injection, growing to greater than 5000 mm^3^ within 5.5–6.5 weeks (Fig. 1C). On MRI, tumors were heterogeneous and sometimes encased or invaded the adjacent kidney (Fig. 1D). Five weeks after injection, tumors ranged in size from 270 to 4675 mm^3^ (2428 ± 1806 mm^3^) (Fig. 1C; Supp Table S1). Of the 18 total rats injected orthotopically with neuroblastoma cells (6 for survival and 12 for surgery), 14 established observable tumors within 35 days, 3 grew tumors in a delayed fashion (meeting the humane endpoint after 8.5–11 weeks), and 1 did not grow any observable tumor within 20 weeks.

**Figure 2:**
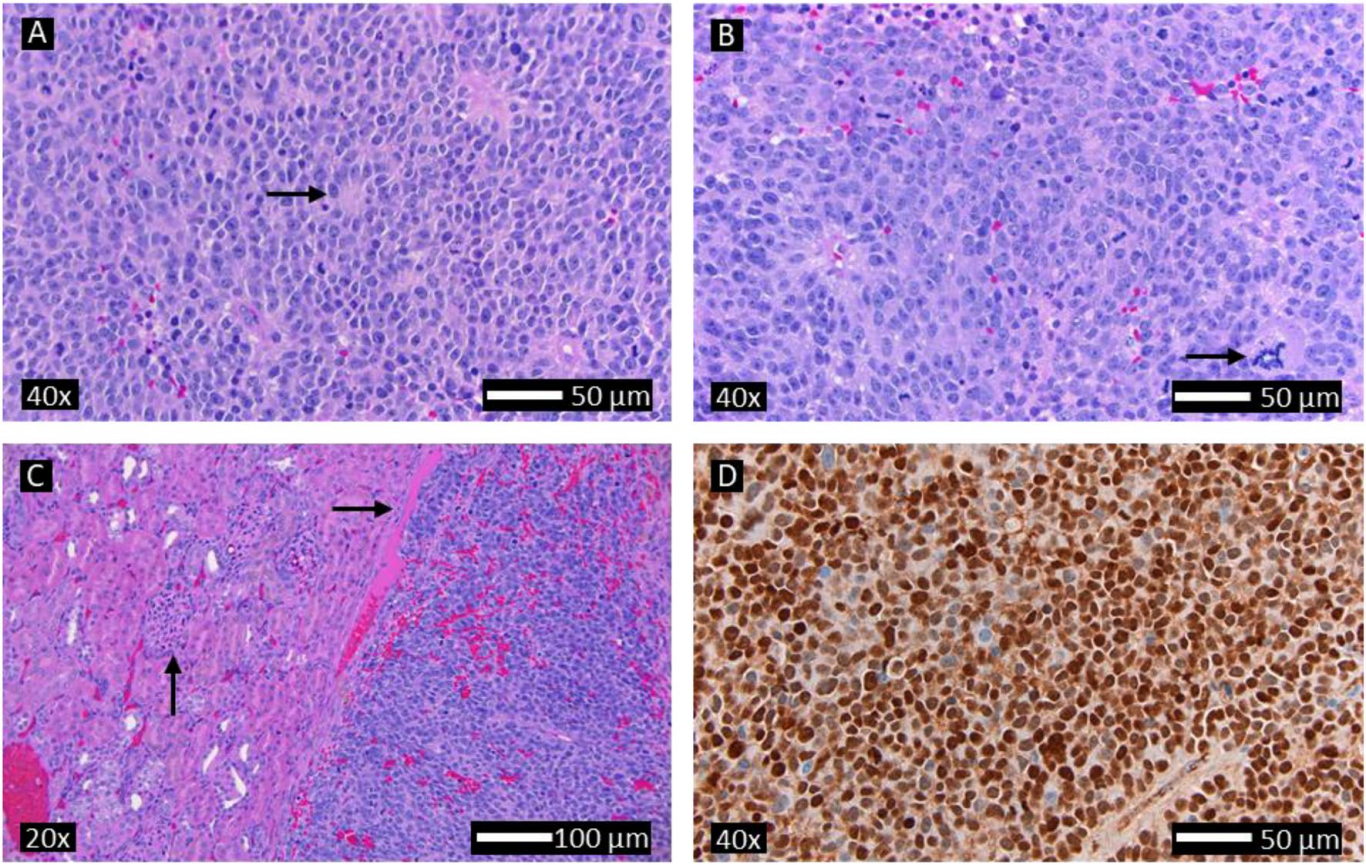
Representative histology of orthotopic xenograft neuroblastoma in the rat (similar in all resected tumors and nodules) showing (**A**) small blue round cells with a Homer Wright rosette indicated by the arrow (H&E), (**B**) multiple mitoses, karyorrhectic figures and prominent nucleoli (consistent with MYC-N amplification), where the arrow indicates anaplastic mitosis (H&E), (**C**) invasion of the renal parenchyma (horizontal arrow shows invasion, vertical arrow shows glomerulus of renal parenchyma), (**D**) and strong expression of PHOX2B with a nuclear pattern (PHOX2B stain). There was some variability in the extent of necrosis, hemorrhage, fibrosis, and PHOX2B staining (all were strongly positive though some had patchy areas of weaker staining).

Rats reached the humane endpoint at a median of 42 days following tumor implantation (81 days of age), at which point tumors were greater than 5000 mm^3^ (Supp Table S1). One to two days prior to the humane endpoint, body condition score decreased from a normal 3 to a deconditioned 2. Weight loss did not occur prior to the endpoint, though in some rats the tumors were above 50 g at endstage, suggesting loss of muscle and/or fat during this time. Difficulty with mobility nor breathing also did not occur until the day of euthanasia.

Tumors were not typically distinguishable on palpation until very near end-stage. On necropsy, tumors were adherent to or invaded the ipsilateral kidney, spleen, stomach, body wall, liver, diaphragm, and ipsilateral ovary (Fig. 1E). Histopathology demonstrated poorly differentiated neuroblastoma with features similar to unfavorable histology seen in human tumors, including high number of mitoses and karyorrhectic figures (> 4% of all cells). Small round blue cell tumors with Homer-Wright rosettes and variable to strong nuclear PHOX2B staining were seen, along with areas of hemorrhage and necrosis, as well as focal invasion of adipose tissue and the renal capsule (Fig. 2).

### Tumor resection

**Figure 3:**
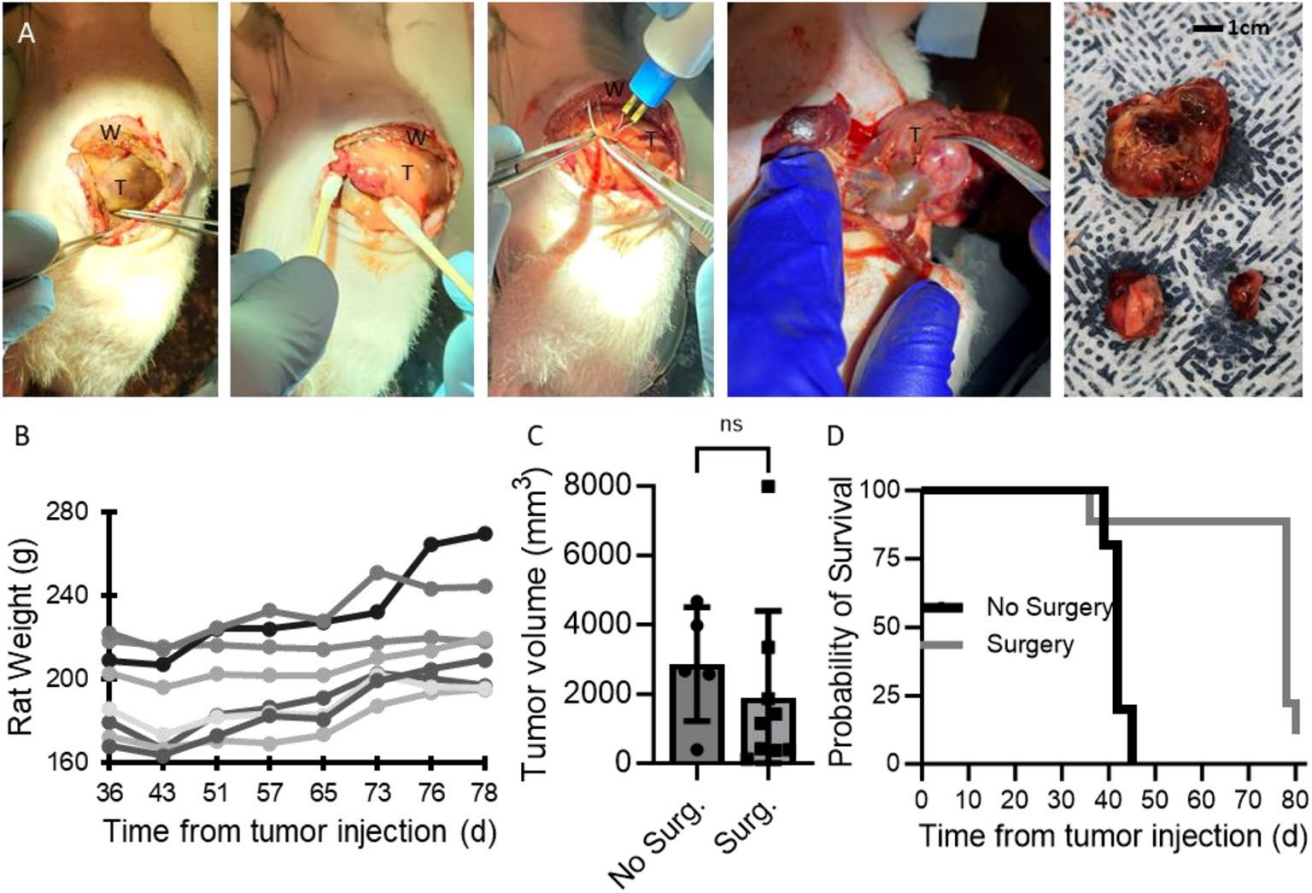
(**A**) Representative images showing surgical resection of neuroblastoma in xenografted rats, showing the left flank incision, early dissection of tumor from surrounding tissue, use of electrocautery, nearly removed tumor, and excised tumor with nodules (T is tumor and W is abdominal wall). (**B**) Weight of rats following surgical resection. (**C**) Tumor size at 5.5 weeks (p = ns) and (**D**) comparative survival of rats without surgery and with surgery (n = 5 for no surgery and n = 9 for surgery groups; p < 0.005).

Five weeks after tumor injection, tumors were resected from rats (n = 9) through a left flank incision, using a combination of sharp dissection and electrocautery (Fig. 3A). One rat with a larger tumor (3347 mm^3^) did not survive resection. Tumors ranged in size (measured after resection) from 123 to 7980 mm^3^ (2146 ± 2439 mm^3^), with four small tumors (123, 370, 394, and 421 mm^3^), three medium tumors (1150, 1420, and 1866 mm^3^), and two large tumors (3347 and 7983 mm^3^) (Supp Table S1). There was a high positive correlation between the tumor volume on MRI and volume measured after resection (slope = 1.2; R^2^ = 0.7). There was no significant difference between the size of tumors at the time of resection for the non-operative (survival) group and the surgical group (Fig. 3C). Adjacent non-contiguous tumor nodules were resected from a few of the rats and confirmed to be neuroblastoma by histology. The remaining 8 rats fully recovered and exhibited no signs of excessive tumor burden.

Following tumor resection, rats lost weight in the first week, regained it, and then plateaued in their weight or continued to gain weight (Fig. 3B). As rats continued to appear healthy (body condition score of 3), with no increased work of breathing and no difficulty ambulating, MRIs were not done until 6 weeks after resection to assess for tumor regrowth. Most rats abruptly developed abdominal distension 6 weeks after resection, at which time MRI was performed, with tumors 1.84 cm to 6.8 cm in largest dimension, with the exception of one rat with no appreciable tumor recurrence. All except two rats (those with no tumor and 1.84 cm tumor) had tumor volume greater than 5000 mm^3^ and/or significant distension and were euthanized (Supp Table S1). The remaining two rats were sacrificed two days later when one also demonstrated abdominal distension. Adhesions were more pronounced than in the initial resection and tumors were often difficult to separate from the spleen and ipsilateral kidney (Fig. 1F). A few tumors involved small bowel or ipsilateral ovary and fallopian tube. There was no correlation between tumor volume at resection and at endstage (R^2^ = 0.1 with or without inclusion of the rat that did not have tumor recurrence) nor between tumor mass at resection and at endstage (R^2^ = 0.03 with and 0.2 without inclusion of the rat without recurrence). There was a very strong correlation between recurrent tumor volume measured on MRI and after resection (slope 1.0; R^2^ = 0.96 with only the recurrent tumors and with all tumors at end-stage).

Rats that underwent tumor resection survived longer than those who did not (median survival 78 days vs 42 days, p < 0.005; Fig. 3D). When rats with small tumors (4 in surgery group, 1 in survival group) were excluded, survival of surgery rats still trended towards longer (median survival 78 days vs 42 days, p = 0.08). While those that underwent tumor resection did not undergo MRIs as frequently and may have met the humane endpoint of tumor volume greater than 5000 mm^3^ earlier than discovered, survival would remained significantly longer in both cases, but to a lesser extent.

## Discussion

Even with the current multimodal therapies, including surgery, high risk neuroblastoma in children remains fatal in 40–50% of patients within 5 years^1,2,22–24^. The challenge remains in providing safe and complete resection, limited by the difficulty of accurate detection of tumor margins, occult nodal disease and the encased neurovascular structures^6^. Sadly, the level of completeness of resection impacts both the patients’ outcomes and their response to consolidation therapy with Dinutuximab^25–27^. The current rodent models prove to be challenging for innovations in surgery as they are largely too small to replicate human tissue (specifically in mouse models), or the disease model is not anatomically similar to the that of the disease in humans. The stand-alone rat adrenal xenograft model was published over 20 years ago^28^. Rats are more typically expensive per animal and fewer rats are housed per cage. Thus, investigators looking to establish a rat xenograft program should weigh the cost-benefits of this model. Additionally, it is important for investigators to consider using the lowest informative species model for studies^29^.

Rats are more typically expensive per animal and fewer rats are housed per cage. Thus, investigators looking to establish a rat xenograft program should weigh the cost-benefits of this model. Additionally, it is important for investigators to consider using the lowest informative species model for studies^29^.

Here, we have developed a novel xenograft rat model of adrenal neuroblastoma using localized injection of a human neuroblastoma cell line, SK-N-BE(2), derived from the metastatic bone lesion of a neuroblastoma in a 2-year-old after multiple rounds of chemotherapy and radiation. Cells suspended in Matrigel were successfully injected directly into the adrenal glands of 5–6-week-old pediatric nude rats (Fig. 1A). Using immunodeficient rodents is common in xenograft models to enable the growth of human tumor cell lines and this method of xenograft implantation is well described in mice but here has been translated to rats^20^. This cell line adequately replicates tumors at the time of surgery, which typically occurs after multiple rounds of induction chemotherapy, then resection, followed by myeloablative consolidative therapy, radiotherapy, and maintenance therapy with biologics such as Dinutuximab^17,22^. Furthermore, derivatives of SK-N-BE(2) cells have been shown to have amplified MYC-N, which is a feature of high-risk disease in humans^3,28^. While this cell line was selected for its performance in the mouse model and for GD2 overexpression for further studies, similar models using cell lines that do not overexpress MYC-N or have different levels of GD2 expression may be attempted. Orthotopic patient-derived xenograft (PDX) models would also be an interesting future direction.

In most rats, tumors grew quickly to greater than 5000 mm^3^ in 35–45 days (Fig. 1C). Three of the eighteen rats implanted with tumor cells grew similar tumors in a delayed fashion while one did not grow a tumor, despite a lack of notable differences in tumor cell injection between those rats and the others. While MRI was used to characterize the growth of tumors in this new rat model, ultrasound could be considered as an alternative though there is more user variability. A strong correlation was seen between tumor volume measured on MRI and after resection both at the initial resection (R^2^ = 0.7) and at end-stage (R^2^ = 0.96 for only the surgery group and for both groups) with slopes very close to 1, indicating the reliability of MRI for determining tumor volume. The majority grew tumors in a predictable timeframe which could also be used to design experiments instead of regular imaging. At least one MRI or ultrasound, however, would be recommended prior to any treatment to determine more exact size. Despite tumor growth, the rats continued to gain weight until meeting humane endpoint criteria (Fig. 1B). MRI demonstrated heterogeneous tumors that abutted, encased, or invaded the adjacent kidney (Fig. 1D). Surgically resected tumors were frequently adherent to neighboring structures including the ipsilateral kidney and spleen, and some rats grew additional tumor nodules. Histopathology was consistent with a poorly differentiated neuroblastoma with unfavorable pathology (Fig. 2) demonstrating small round blue cell tumors with Homer-Wright rosettes (Fig. 2A). Tumors expressed diffuse PHOX2B staining in a nuclear pattern (Fig. 2D), which is a sensitive and specific (versus other pediatric small round blue cell tumors) marker of neuroblastic tumors^30^. There were also large areas of hemorrhage and necrosis with focal invasion of adipose tissue and kidneys (Fig. 2C). Tumors also had a high mitosis/karyorrhexis index, indicating unfavorable histology (Fig. 2B). Further work will include assessment of MYC-N overexpression in the tumors (which has been shown in the SK-N-BE(2) cell line) and Ki67 proliferation index in both initial and recurrent tumors. While risk stratification in humans incorporates clinical features such as age, which cannot be truly replicated in rodent models, the poorly differentiated phenotype with unfavorable histology likely recapitulates aggressive disease, which would benefit most from improved therapy. Cumulatively, the radiographic and histopathologic features are similar to those seen in humans.

Thirty-five days after tumor injection (70–77 days of age), tumors were of sufficient size for surgical resection (Fig. 1C). In pediatric neuroblastoma surgery, neighboring organs with small disease deposits are typically left in place to preserve function and portions of the tumor must be removed sequentially to preserve vital structures such as vasculature. In this rat model, however, gross invasion of neighboring organs including the kidney was not evident, so no macroscopic disease was left behind, and tumors could be removed intact (though nodules were occasionally removed separately). Consistent with human surgery for neuroblastoma, renal capsule was stripped off the kidney with the tumor if there was invasion, but minimal to no functional renal parenchyma was resected. All grossly visualized tumor was resected from each rat. Surgical resection was successful in 8 of 9 rats and those rats recovered well from surgery, though 7 tumors recurred within 6 weeks. There was no correlation between tumor volume nor mass at resection and at end-stage. Notably, surgical resection increased the lifespan of neuroblastoma rats following tumor injection (from a median of 42–78 days; p < 0.05; Fig. 3D). Earlier imaging may have suggested an earlier humane endpoint in the surgical group, though likely only by 1–2 weeks based on the growth curve of the initial group (with the increase in lifespan remaining significant). In future work, all rats will be imaged beginning within 3 weeks of tumor injection and surgical resection to assess tumor burden. While the purpose of this study was to demonstrate feasibility of this rat model and extension of lifespan following resection, future work could include repeated excision of tumors to assess further efficacy and resection in a more challenging operative field due to additional adhesions and scar tissue, as well as other changes secondary to treatment.

The increased size of the abdominal organs and tumor, as well as the adrenal location of the xenograft in this novel rat model is critical for enabling future surgery-based animal studies. This novel surgical neuroblastoma model will enable studies of the combined impact of surgery with pre-, peri-, and post-operative treatments, including chemotherapy, photodynamic therapy, dinutuximab, and novel therapeutics. Additionally, this model will enable in vivo assessment of intraoperative contrast agents to enhance resection of neuroblastoma.

## Conclusions

High-risk neuroblastoma remains fatal in 40–50% of children within 5 years, despite a combination of surgical resection and other therapeutics. As such, a model that accurately recapitulates the disease and enables a combination of those treatments is critical for the development of novel treatments. Here, we have developed an orthotopic xenograft rat model of neuroblastoma with pathology consistent with poorly differentiated human neuroblastoma with unfavorable features, including small round blue cell tumors with Homer-Wright rosettes, diffuse PHOX2B staining in a nuclear pattern, and a high mitosis/karyorrhexis index. We have demonstrated the ability to perform surgical resection of neuroblastoma on live animals, with nearly all surviving the procedure. Rats that underwent surgery survived significantly longer than rats that did not undergo resection, though tumors recurred in 7 of 8 rats within 6 weeks. This novel rat model of neuroblastoma can be used in the future to assess efficacy of surgical resection in combination with other treatments.

### Supplementary Information

Supplementary Table 1.

## Data Availability

The datasets used and/or analyzed during the current study are available from the corresponding author on reasonable request.
