# Supplementary material for: Survival following complete resection of neuroblastoma in novel orthotopic rat xenograft model
Source: Sci Rep. 2023 Nov 18;13:20214. doi: 10.1038/s41598-023-47537-3 (PMC10657433; doi:10.1038/s41598-023-47537-3)
Supplement: Supplementary file 1 — Supplementary Table 1. [file 41598_2023_47537_MOESM1_ESM.docx]

**Supplemental Table 1.** Data for each rat included in the surgery and non-surgery groups (n=9 and n=5, respectively), including tumor volumes (by MRI and/or caliper measurement), tumor weights, duration of surgery, and survival after tumor cell injection.

| **Rat ID** | **Cohort** | **Tumor Volume**  **(4w after tumor injection; MRI; mm^3^)** | **Tumor Volume**  **(5w after tumor injection;**  ***ex vivo* for surgery group & MRI for no surgery group; mm^3^)** | **Tumor Weight at Resection (grams)** | **Duration of Surgery (minutes)** | **Survival after tumor injection**  **(days)** | **Tumor Volume at End Stage by MRI (mm^3^)** | **Tumor Volume at End Stage *ex vivo* (mm^3^)** | **Tumor Weight at End Stage (grams)** |
| --- | --- | --- | --- | --- | --- | --- | --- | --- | --- |
| 881-0 | Surgery | 356 | 123 | 0.1 | 39 | 80 | 2,157 | 1,638 | 1.1 |
| 881-1 | Surgery | 669 | 370 | 0.2 | 42 | 78 | 33,196 | 26,158 | 21.2 |
| 882-0 | Surgery | 3,956 | 7983 | 3.6 | 51 | 80 | 0 | 0 | 0 |
| 882-1 | Surgery | 4,806 | 3347 | 4.0 | 100 (did not survive) | 36 |  |  |  |
| 883-0 | Surgery | 773 | 1150 | 0.9 | 31 | 78 | 141,855 | 156,933 | 72.7 |
| 884-1 | Surgery | 538 | 421 | 0.3 | 47 | 78 | 18,735 | 19,890 | 9.4 |
| 885-0 | Surgery | 2,227 | 1,866 | 2.1 | 55 | 78 | 70,037 | 56,889 | 26.3 |
| 885-1 | Surgery | 478 | 394 | 1.1 | 23 | 78 | 127,714 | 152,460 | 63.6 |
| 886-1 | Surgery | 1,187 | 1,420 | 0.85 | 36 | 78 | 145,293 | 126,672 | 53.7 |
| 638-0 | No surgery | 2,400 | 4,675 |  |  | 39 | 6,510 | 7750 | 9.3 |
| 638-1 | No surgery | 27 | 270 |  |  | 45 | 8,820 | 5,465 | 7.5 |
| 639-0 | No surgery | 594 | 2,574 |  |  | 42 | 11,745 | 9,744 | 6.4 |
| 639-1 | No surgery | 1,989 | 2,669 |  |  | 42 | 7,176 | 22,800 | 10.3 |
| 640-1 | No surgery | 2,574 | 3,978 |  |  | 42 | 6,930 | 16,751 | 8.7 |
